# Research on the Surface Deformation, Fault Rupture, and Coseismic Geohazard of the 2022 Luding Mw 6.8 Earthquake

**DOI:** 10.3390/s23249875

**Published:** 2023-12-16

**Authors:** Yiling Lu, Yinghui Yang, Li Zeng, Wanfu Xu, Jiawei Song, Xiaoyun Li

**Affiliations:** 1 School of Architecture and Civil Engineering, Chengdu University, Chengdu 610106, China; luyiling@cdu.edu.cn (Y.L.);; 2 State Key Laboratory of Geohazard Prevention and Geoenvironment Protection, Chengdu University of Technology, Chengdu 610059, China

**Keywords:** Luding earthquake, coseismic InSAR deformation, faulting model inversion, coseismic geohazard

## Abstract

An Mw 6.8 earthquake occurred in Luding County, Ganzi Tibetan Autonomous Prefecture, Sichuan Province, on 5 September 2022. This seismic event triggered numerous coseismic geohazards in the seismic zone. In this study, the ascending- and descending-track synthetic aperture radar (SAR) images observed by the Sentinel-1A satellite are utilized to extract the coseismic surface deformation of the Luding earthquake. Subsequently, a faulting model is estimated based on the elastic dislocation theory, under the constraint of the InSAR observation. Additionally, the POT technique was employed to detect coseismic geohazards. High-spatial-resolution optical remote sensing images served to validate the reliability of the detection results. The coseismic interferometric synthetic aperture radar (InSAR) deformation field indicated a maximum deformation of ~190 mm and ~140 mm along the ascending and descending tracks, respectively. The estimated best-fitting faulting model suggests that the optimal seismogenic fault strike and dip angles are 169.3° and 70°, respectively. The fault slip predominantly exhibits left-lateral strike-slip characteristics and is concentrated at depths of 3–12 km. The estimated maximum fault slip was 2.67 m, occurring at a depth of 7 km. The pixel offset tracking (POT) result derived from the pre- and post-earthquake SAR images found a total of 245 medium- to large-scale coseismic geohazards, with a verification rate from optical images exceeding 64%. The distribution of these geohazards is notably dense within the significant fault rupture segment. Geohazards on the fault hanging wall are densely packed, whereas landslides along the Dadu River’s fault footwall are also notably frequent.

## 1. Introduction

**Figure 1:**
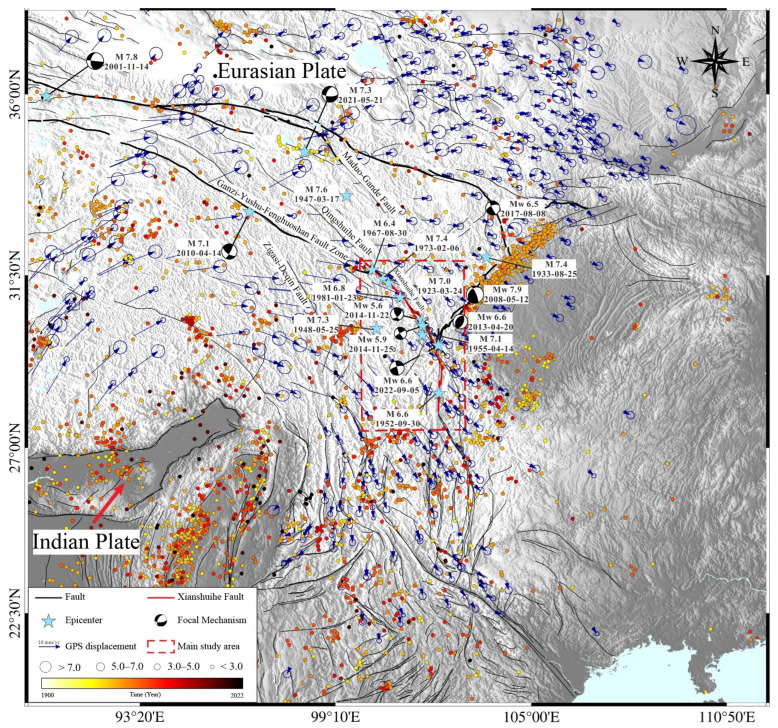
Reginal geological background. The thick, black, solid lines denote the active faults while thin, black, solid lines indicate the active fault surface trace. The blue arrows show the GPS horizontal displacement. The circles are the instrumental earthquakes, the blue star indicates the epicenter, the color of the circle presents the focal depth, and the black beach balls show the mechanism of historical earthquakes. The red dotted rectangle shows the extent of the main study area and the red solid lines indicate the Xianshuihe fault.

On 5 September 2022, at 12:52 p.m. (Beijing Time), an earthquake with a moment magnitude (Mw) of 6.8 struck Moxi Town, Luding County, Ganzi Tibetan Autonomous Prefecture, Sichuan Province. The epicenter was situated at coordinates 29.59° N and 102.08° E, approximately 7 km from Moxi Town and 20 km from Gongga Mountain (Figure 1). As of the most recent data, the Luding Mw 6.8 earthquake resulted in 93 fatalities and 25 individuals remain unaccounted for [1].

Following the earthquake, preliminary reports from the China Earthquake Administration indicated a focal depth of approximately 16 km, a duration of around 20 s, and a maximum intensity of IX degrees. The earthquake was classified as a main-aftershock type [2]. Concurrently, the focal mechanisms reported by China Earthquake Networks, the United States Geological Survey (USGS), and other organizations suggest that the Luding earthquake was a high-dip strike-slip event. The causative fault has been identified as the Kangding to Shimian segment of the Xianshuihe fault, situated proximal to Moxi town (Figure 1). The Xianshuihe fault zone, spanning over 400 km, delineates the southern boundary of the Bayan Har block within the Tibetan Plateau. This fault zone holds significance in accommodating the relative motion between the Indian and Eurasian plates. Additionally, it stands as one of China’s most seismically active regions. Major earthquakes, including the Diexi Mw 7.0 in 1923, Litang Mw 7.3 in 1948, Kangding Mw 7.1 in 1955, Luhuo Mw 7.4 in 1973, and Yushu Mw 7.1 in 2010, have all manifested along this fault line, leading to substantial human and economic losses [3,4,5,6].

The magnitude of the 2022 Luding earthquake, coupled with the strategic location of key water systems, like the Dadu River and the region’s hazardous terrain and intricate river basins, instigated a multitude of landslides, collapses, and other geohazards. This subsequently led to notable infrastructural damage to roads and houses. The region’s intricate topography, compounded by compromised transport conditions post-earthquake and a sparse distribution of near-field seismic wave stations, often impedes the swift acquisition of coseismic surface deformations and post-earthquake geohazard distributions. To elucidate the earthquake mechanism and associated geohazards, Fan et al. (2022) employed post-earthquake optical satellite remote sensing images for a prompt identification and interpretation of landslides within the seismic zone, successfully mapping the distribution of coseismic landslides [7]. However, the imagery utilized by Fan et al. (2022) encompassed only a segment of the seismic zone, thereby curtailing a comprehensive understanding of post-earthquake geohazards across the entire region.

To elucidate the focal mechanism of the 2022 Luding earthquake and its consequent effect on geohazards, the interferometric synthetic aperture radar (InSAR) technique is employed to derive the coseismic surface deformation field associated with the earthquake. Subsequently, a coseismic fault rupture model is determined, guided by the constraints of the aforementioned surface deformation. Using pixel offset tracking (POT) between pre- and post-earthquake SAR images, coupled with local topographical and geomorphological data, medium to large landslides induced by the earthquake are identified. Optical remote sensing images provide validation for these findings. Lastly, the correlation between the coseismic fault slip and the resultant geohazards is explored.

## 2. Regional Tectonic Background

The subduction and convergence of the Indian plate into the Eurasian plate instigated the southeastward extrusion of the Qinghai Tibet Plateau’s crustal materials [8]. Consequently, numerous active, large-scale fault zones emerged in and around the Tibetan Plateau. These zones significantly regulate the strike slip and thrust arising from the plates’ relative movement [9]. Positioned at the northeastern intersection of the Bayankala block and Sichuan–Yunnan block, the Xianshuihe fault zone constitutes the central segment of the Xianshuihe fault system (Figure 1). Spanning approximately 400 km with a predominant strike angle ranging between 320–330°, this zone displays sinistral strike-slip dynamics [10]. The fault zone originates from the Ganzi’s Kasu area in the northwest, extends southeastward through Luhuo, Daofu, Kangding, and Moxi, and culminates near the Anshunchang region of Asbestos [11]. Furthermore, the southern segment of the Xianshuihe fault zone, in conjunction with the Longmenshan fault zone to the southeast and the Anninghe fault zone to the south, shapes the “Y-shaped” fault configuration in western Sichuan [12].

To understand the activity of the Xianshuihe fault, researchers employed various methods to observe the sliding rate across its sections. These observations aimed to discern the temporal and spatial characteristics of fault activities, and to gauge the seismic risk associated with each section. Using fault landforms, Qian et al. (1988) characterized the activity of the Xianshuihe fault zone since the Holocene, revealing distinct long-term slip rates between the northern and southern ends. Specifically, the northern Qianning section exhibited a rate of approximately 15 ± 5 mm/year, while the Kangding fault’s southern section was estimated at 5.5 mm/year [13]. Utilizing the extant research data, Wen et al. (2000) conducted a stratified analysis of the seismic rupture attributes of the Xianshuihe–Anninghe–Zemuhe fault zone and performed a segmented examination of fault activities. The motion rates observed for the central fault were 1–2 mm/year for the Yalahe section (eastern branch), 5–8 mm/year for the Selaha section (central branch), and 3.6–8.5 mm/year for the Zheduotang section (western branch). Additionally, the Moxi fault displayed a slip rate of around 6–9.9 mm/year [14]. Incorporating the regional field survey data, trench excavation, and historical seismic analysis, Liang et al. (2020) assessed a slip rate of 5 mm/year for the Zheduotang section and 8.4 mm/year for the Luhuo section, marking the inaugural determination of fault-zone activity variations over disparate periods [15]. From a geodesic perspective, Meng et al. (2008) used a two-dimensional spiral dislocation model with GNSS data to deduce slip rates of 11.4 mm/year for the northwest section and 8.4 mm/year for the southeast section of the Xianshuihe fault [16]. Analyzing the Sentinel-1A/B data for 2014–2019, Qiao et al. (2020) discerned that a shallow creep phenomenon permeated the Xianshuihe fault-zone sections. The inversion findings suggest slip rates of 3.3–7.8 mm/year for the Qianning–Daofu section and 16.3–19.8 mm/year for the Kangding section, with the entire section’s locking depth ranging between 7.6–18.5 km [10].

The Xianshuihe fault zone displays a notable slip rate and pronounced activity in each of its sections, resulting in significant geohazards throughout the entirety of the fault [14]. The historical seismic catalog data reveal the occurrence of at least 10 earthquakes with a magnitude of Mw 7.0 or higher along this fault (Figure 1). Furthermore, in-depth tstudies indicate that, following the 2008 Wenchuan Mw 7.9 earthquake, the 2013 Lushan Mw 6.6 earthquake, and the 2017 Jiuzhaigou Mw 6.5 earthquake, there has been a marked increase in the static Coulomb failure stress within the Kangding region [17,18,19]. Such an increase can predispose the region to major seismic events. Notably, despite the Kangding region experiencing two successive moderate earthquakes (Mw 5.9 and Mw 5.6) in November 2014, these seismic activities only mitigated a fraction of the accumulated strain on the fault, suggesting its latent potential for producing more powerful earthquakes [12,20,21].

## 3. InSAR Coseismic Deformation Field of the Luding Earthquake

**Figure 2:**
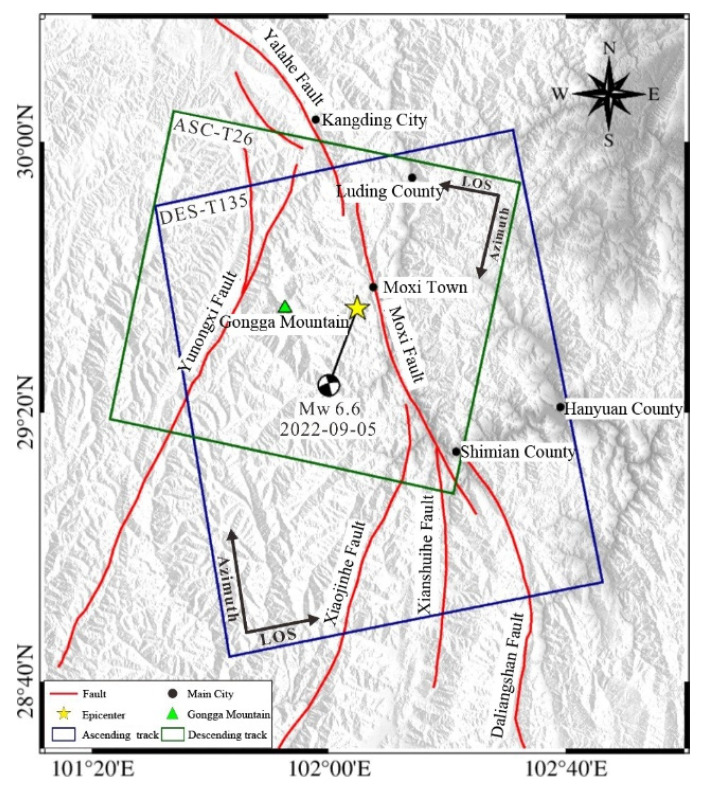
Coverage of the SAR image. The red solid lines denote the main faults and the black dots show the main cities in the region. The yellow star denotes the epicenter of the 2022 Luding earthquake. The green triangle shows the location of Gongga Mountain. The blue rectangle represents the ascending-track coverage and the green rectangle shows the descending-track coverage.

The Luding earthquake occurred in a high-altitude mountainous region characterized by a low population density, intricate topography, abundant vegetation, and an expansive coseismic area. Traditional methods, such as field investigation, GPS, and leveling, pose challenges in obtaining precise surface deformation information from such areas. Thus, pre- and post-earthquake C-band SAR images (Figure 2), captured by the European Space Agency (ESA) Sentinel-1A satellite, were procured. The specific image parameters are presented in Table 1. The InSAR technique facilitated the extraction of the coseismic deformation field pertaining to this earthquake.

First, two radar images spanning the deformation period were used for InSAR interference processing, and the reference ellipsoid phase was removed. Then, external DEM registration sampling was conducted in the image interference region, and the corresponding topographic phase information was calculated according to the interference baseline and the registration external DEM elevation information; then, the topographic phase information was deducted from the interference phase. Finally, the differential interference phase consisting mainly of the deformation signal was obtained (the effects of the atmospheric delay phase, system noise phase, etc., were not considered here). Inversely, the deformation phase can be defined as follows:

φ_def_ = φ − φ_ref_ − φ_top_ (1)

where φ_def_ is the deformation phase, φ is the interference phase, φ_ref_ is the reference ellipsoid phase, and φ_top_ is the topographic phase [22].

**Figure 3:**
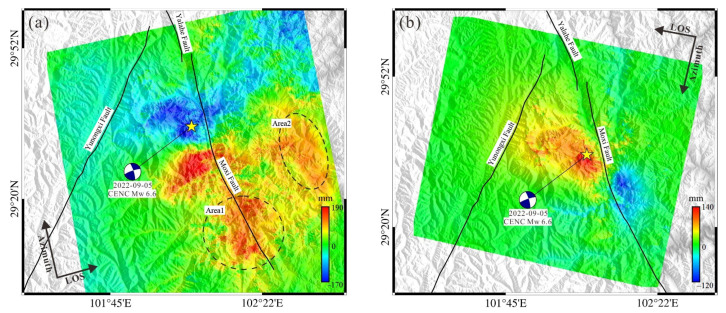
InSAR coseismic deformation of ascending (**a**) and descending (**b**) tracks. The black solid lines show the main faults in the region. The blue beach ball indicates the mechanism of the 2022 Luding earthquake and the yellow star shows the epicenter. The color presents the deformation magnitude.

For the data processing step, GAMMA [23] software (GAMMA-2020 software) was utilized. As the epicenter was situated at the confluence of two landscape images on ascending tracks, both images were combined to maintain the deformation field integrity. The SRTM-DEM data (with a spatial resolution of 30 m) released by the National Aeronautics and Space Administration (NASA) were employed to simulate and eliminate the InSAR topography phase component. Given the region’s dense vegetation, the interferogram underwent Gaussian filtering to enhance the interferometric signal-to-noise ratio. Additionally, the minimum cost flow (MCF) method was used for phase unwrapping [22]. Acknowledging that satellite orbital data inaccuracies might produce residuals, high-coherence far-field data from the fault were harnessed to construct a bilinear orbital trend surface, thus offsetting the orbital error. Following the phase-to-deformation conversion, the DEM data facilitated geocoding, ultimately extracting the coseismic InSAR deformation field of the satellite along both ascending and descending tracks covering the Luding earthquake’s seismic region (Figure 3).

Figure 3 illustrates the coseismic surface deformation of the Luding earthquake predominantly situated near the Moxi fault. The major deformation axis is positioned in the NEE-SWW direction, with the deformation approximately symmetrically distributed on both the northwest and southeast flanks of the fault. The satellite’s line of sight (LOS) deformation field on each side of the fault exhibits opposing deformation directions. Upon further analysis, considering the regional tectonic background, it is inferred that the earthquake might represent a high-dip strike-slip seismic event. The ascending-track InSAR deformation depicted in Figure 3a reveals the southwest flank of the fault is characterized by movement away from the satellite, with a peak deformation magnitude reaching ~170 mm. Conversely, the northwest side indicates surface movement toward the satellite, exhibiting a maximum deformation of ~190 mm. It is essential to note that, due to de-correlation, atmospheric influences, and other variables, no substantial and dependable surface motion signal was discerned in the fault’s eastern region from the ascending-track InSAR. Certain local anomalies within the black dashed ellipse (Figure 3a) are likely attributable to a combination of interference de-correlation, phase unwrapping errors, and atmospheric disturbances. In comparison, the descending-track InSAR, presented in Figure 3b, boasts a superior signal-to-noise ratio. The western side of the fault predominantly presents movement toward the satellite, with a peak deformation magnitude of ~140 mm, whereas the southeast side displays movement away from the satellite, with a deformation magnitude peaking at ~120 mm. The delineated InSAR deformation field distribution aligns closely with the patterns observed in other high-dip strike-slip earthquakes oriented in the proximate south–north direction [24,25,26].

## 4. Inversion of the Fault Rupture Model of the Luding Earthquake

The seismogenic mechanism solution of the Luding earthquake fault was derived using the ascending- and descending-track InSAR deformation, grounded in the theory of the elastic half-space dislocation model [27]. This was conducted to improve our understanding of this earthquake’s seismogenic mechanism. The inversion procedure encompassed two primary steps. Initially, the probable location of the seismogenic fault was pinpointed using InSAR observations and the tectonic background data. Subsequently, the fault plane was discretized using a coarse grid. Employing the constraint of the surface deformation data, the simulated annealing nonlinear constraint algorithm [28] was adopted to identify the optimal fault geometric parameters. These parameters included the fault strike, dip angle, depth, and rake angle. Concurrently, the magnitude of the fault slip was ascertained via the linear least square inversion. Following this, with the best-fitting fault geometric parameters established, the fault geometry model was delineated. Upon refining and discretizing the fault plane, the detailed motion model of the fault plane was ultimately derived through inversion, constrained by InSAR observations [29,30].

### 4.1. De-Resample of InSAR Deformation

**Figure 4:**
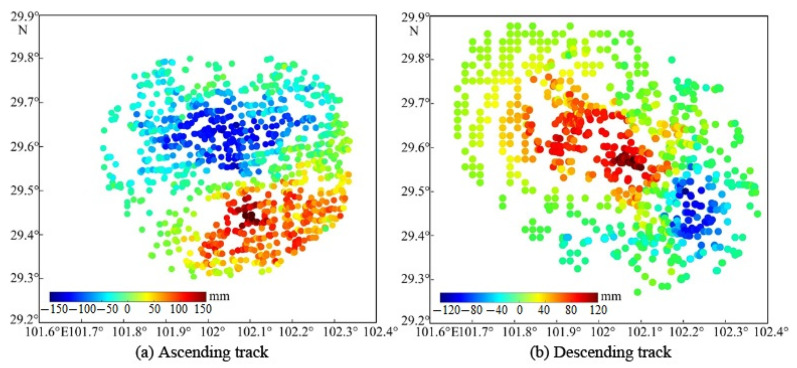
The sampled InSAR deformation outcomes for ascending (**a**) and descending (**b**) tracks. The colored dots show the location of the samples, and the colors indicate the deformation magnitude.

The coseismic surface deformation data, acquired through the InSAR technique, were voluminous and spatially continuous, complicating their direct application to the fault parameter inversion. Additionally, the InSAR images of certain regions exhibited a subpar quality, predominantly attributed to InSAR interference noise, rendering them unsuitable for fault parameter inversion purposes. To address this issue, the deformation field was initially masked using the coherence coefficient, eliminating observations with an interferometric coherence below 0.3. Subsequently, the quadtree algorithm was employed to de-resample the masked data from both the ascending and descending tracks of the InSAR deformation [31], ensuring the preservation of pertinent deformation data. Consequently, 1473 and 1094 InSAR samples were retained for the ascending- and descending-track observations, respectively, as depicted in Figure 4.

### 4.2. Search for Best-Fitting Fault Geometric Parameters

Assuming that the fault plane slides uniformly, the surface deformation caused by the fault dislocation at a monitoring point can be expressed as:

u = Gs(2)

where u indicates the surface deformation of the surface point, the fault slip vector, s, is the combination of the strike slip and dip slip of the fault, and G is a nonlinear expression function of the fault location, length, width, depth, dip angle, and strike angle.

After optimizing the geometric parameters of the fault, the spatial fault plane could be constructed and then discretized by grid; the slip of any sub-fault affected the three-dimensional deformation of the surface monitoring base station. If the covariance matrices of the surface deformation parameters are defined as C, the least square solution of Formula (2) can be estimated as follows:

s = [G^T^C^−1^G]^−1^G^T^C^−1^u (3)

Based on the coseismic InSAR deformation field distributions of both the ascending and descending tracks of the Luding earthquake, the fault plane’s initial dimensions were established as 52 km × 25 km. Incorporating the regional tectonic background information and focal mechanism solutions from GCMT (https://www.globalcmt.org/, accessed on 3 October 2022) and USGS (https://www.usgs.gov/, accessed on 3 October 2022), the preliminary values for the fault strike angle, dip angle, depth, and rake angle were set at 160°, 80°, −10.0 km, and 0°, respectively. Subsequent optimizations for these parameters spanned ranges of 140–180°, 60–90°, −30–0 km, and −30–30°, respectively. Additionally, the fault plane was segmented into coarse 3 km × 3 km grids. Utilizing the simulated annealing algorithm, with the objective of minimizing the model’s residual error, the optimal fault geometric parameters for the Luding earthquake were discerned as follows: a strike angle of 169.3°, a dip angle of 70°, and an average rake angle of −8.5° (refer to Table 2).

### 4.3. Inversion of Fault Slip Distribution

**Figure 5:**
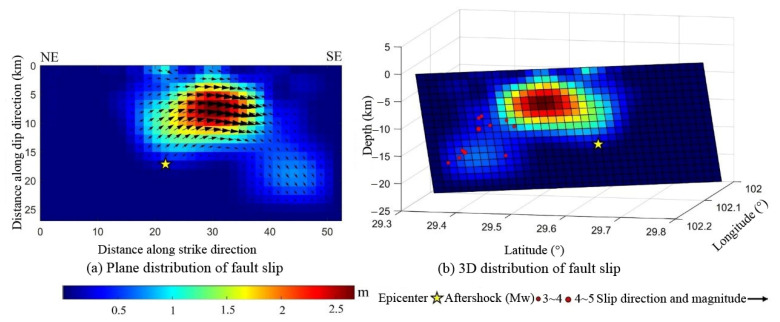
Estimated coseismic fault slip model. (**a**) The plane distribution of the fault slip. (**b**) Three-dimensional view of the fault slip. The yellow star denotes the epicenter; the colors indicate the slip magnitude. The black arrows in (**a**) indicate the slip direction and magnitude. The red dots in (**b**) show the magnitude of the aftershocks.

The slip plane underwent further refinement and was discretized into a 1.5 km × 1.5 km grid based on the determined geometric parameters of the fault plane. Subsequently, the motion vector of the fault plane was ascertained considering the constraints from both the ascending and descending tracks of the InSAR deformation. These findings are illustrated in Figure 5. The analysis revealed that the coseismic fault rupture associated with the Luding earthquake predominantly spanned a depth of 3–12 km below the surface, extending 21 km along the fault strike. The dislocation primarily exhibited left-lateral strike-slip characteristics, with an average rake angle of −8.5°. The position of the maximum slip was pinpointed at a depth of 7 km, exhibiting a magnitude of 2.67 m. Based on the inferred fault rupture model, the moment magnitude of the Luding earthquake was estimated at Mw 6.72 (Table 2). This value was marginally higher than the figure published by the USGS, but aligned more closely with the report by GCMT. Table 2 further highlights the discrepancies between the present study, the findings obtained by the USGS, and reports by GCMT in terms of the fault strike and magnitude. Such differences may largely stem from the distinct high dip angle traits of the fault, coupled with the fact that the findings obtained by the USGS rely on far-field seismic waveform data. Given the absence of a detailed crustal structure model and proximate observation stations, the inversion results from the USGS might present significant uncertainties [32].

Previous studies have determined the slip distribution of this earthquake using different seismological methods [33,34]. The comparison of the coseismic slip results between this paper and these two studies are shown in Table 3. It can be seen in Table 3 that, firstly, the fault characteristic and fault rupture range are relatively consistent; secondly, the seismogenic fault length along the fault strike is shorter than the others; and the maximum slip magnitude is relatively larger than the others, but located at the same depth as the result presented by Li et al. (2022) [33].

### 4.4. Forward Modeling and Residual Analysis of the InSAR Deformation Field

**Figure 6:**
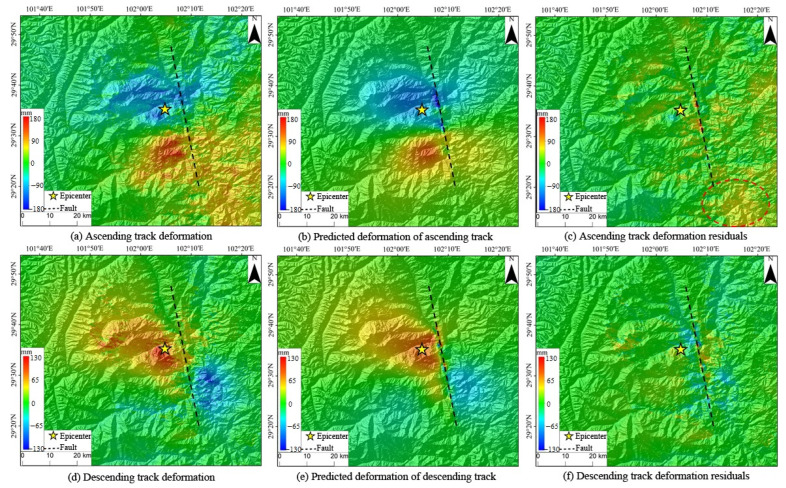
The observed and predicted coseismic InSAR deformation fields. Observed deformation values of ascending (**a**) and descending (**d**) tracks. Predicted deformation of ascending (**b**) and descending (**e**) tracks. Deformation residuals of ascending (**c**) and descending (**f**) tracks. The black dashed line signifies the surface projection of the fault, the yellow star indicates the epicenter, and the colors denote the deformation distribution and magnitude. The red dashed circle in (**c**) shows the residual signals in the southeastern quadrant of the far-field InSAR along the ascending track.

To authenticate the trustworthiness of the inversion results obtained in this study, the inversion fault rupture model was employed to forward calculate the deformation data from both the ascending and descending tracks associated with this earthquake. These data were then juxtaposed against observational values (Figure 6, where the black dashed line signifies the surface projection of the fault). As delineated in Figure 6, both the forward-calculated ascending- and descending-track deformation values align remarkably with the original observational data in terms of the deformation distribution and magnitude, underscoring the credibility of the inversion fault rupture model. Examining the residual diagram reveals that the residuals for most areas are within the −5 mm–5 mm range. Notably, larger residuals are predominantly localized within the fault region. This earthquake induced substantial surface movements and inflicted damage to the proximal fault zone, leading to a pronounced InSAR interference de-correlation within this area. Such occurrences can explain the variance between the observational and forward modeling data. In addition, anomalous residual signals were detected in the southeastern quadrant of the ascending-track InSAR’s distal region. Given the considerable distance of this region from the fault, it was postulated that these signals originated from a fusion of atmospheric delay errors and InSAR interference de-correlations.

## 5. Identification of the Coseismic Geohazards

### 5.1. Pixel Offset Tracking Deformation Extraction

Unlike visible spectral remote sensing imaging, a radar wave’s robust penetration ensures that its observational data remain less influenced by cloud and fog obstructions, enhancing the precise identification of coseismic geological hazards. Nonetheless, the traditional D-InSAR technique often encounters an interference de-correlation, especially given the significant surface deformation in the epicenter area. This characteristic renders it less optimal for detecting and identifying coseismic geological hazards. Thus, the POT technique, known for its superior adaptability to extensive surface deformation characteristics [35,36,37], was employed to delineate the coseismic surface deformation in the epicenter and pinpoint coseismic geohazards.

The POT methodology grounded in SAR images involves intensity and coherence tracking approaches. The former necessitates distinct terrain texture information within the images, while the latter demands that the test area maintains notable coherence [38]. Given that the pronounced surface deformation induced by earthquakes often results in interference incoherence, the intensity tracking approach emerges as more appropriate for processing the data in the epicenter region. This method operates as follows: preliminary coarse registration data are derived from dual-track SAR images. Parameters, such as the image search window and step, are defined, and the corresponding points between the two images are identified through the normalized cross-correlation function (NCC). Subsequently, surface displacement is deduced based on the radar coordinate shifts of these points. Importantly, an appropriate search window and step size are vital for the offset computation of SAR intensity images. Oversized search windows can severely hamper computational efficiency, while undersized ones can yield excessive mismatching points. Through iterative testing, this study adopted a search window of 150 pixels × 30 pixels, striking a balance between reducing mismatch occurrences and ensuring computational feasibility. Notably, given the collective impacts of factors, like image spatial resolution, deformation sensitivity, and registration precision, the SAR-POT approach exhibited reduced sensitivity to minute geohazard targets. Predominantly, the identified geohazards were of a medium to large scale [37,39,40].

### 5.2. Analysis of the Coseismic POT Results

**Figure 7:**
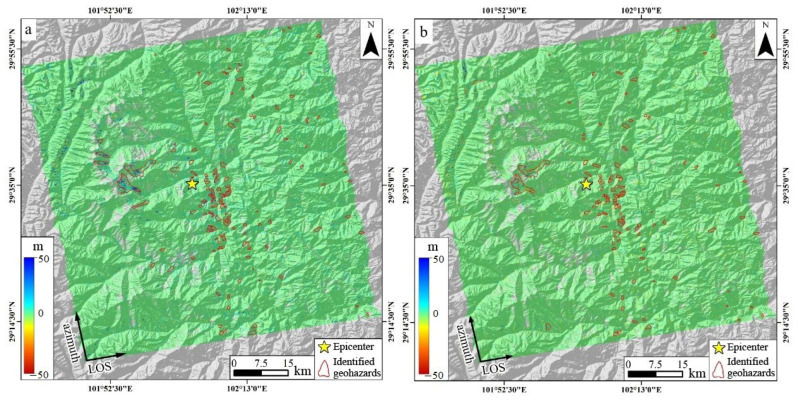
POT-derived deformation along the LOS (**a**) and azimuth directions (**b**) based on the ascending-track SAR images. The yellow star denotes the epicenter, the red closure lines indicate the identified geohazards, and the color of the geohazard areas show the deformation magnitude.

**Figure 8:**
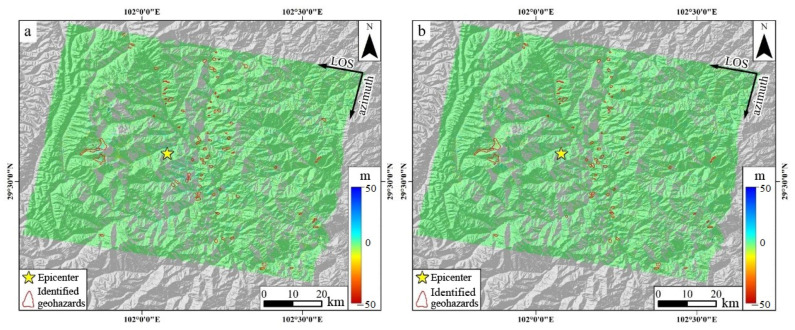
POT-derived deformation along the LOS (**a**) and azimuth directions (**b**) based on the descending-track SAR images. The yellow star denotes the epicenter, the red closure lines indicate the identified geohazards, and the color of the geohazard areas show the deformation magnitude.

Utilizing the pre- and post-earthquake SAR images acquired by Sentinel-1A, the InSAR deformation field along the line-of-sight (LOS) and azimuth directions was extracted from both satellite ascending and descending tracks using the referenced POT technique (Figure 7 and Figure 8). In these figures, the color variations depict the motion magnitude. For the LOS direction deformation, blue signifies the ground surface moving away from the satellite, whereas red indicates a movement towards the satellite (Figure 7a and Figure 8a). Conversely, in the azimuth direction deformation, blue corresponds to a motion aligned with the satellite’s flight, and red represents the opposite movement (Figure 7b and Figure 8b).

It is imperative to acknowledge that the Luding earthquake fault rupture did not propagate directly to the surface, resulting in a comparatively modest magnitude of coseismic static surface deformation (Figure 3). The resolution of the Sentinel satellite image in the IW format stands at 14.0 m × 2.3 m (azimuth direction × LOS direction). The present consensus posits that the deformation recognition accuracy of the intensity correlation POT technique for SAR images is approximately one-tenth of its image resolution [38,41]. This translates to a deformation accuracy of POT being approximately 1.4 m × 0.2 m, a value substantially higher than the coseismic surface deformation magnitude of the Luding earthquake. As a result, InSAR observations from both the ascending and descending tracks could not capture the coseismic surface deformation data associated with this earthquake. Nevertheless, surface deformations induced by coseismic geohazards, notably landslides, typically range in the order of ten meters or even tens of meters. Leveraging the POT technique to extract such large-scale surface deformations is highly viable.

The analysis of the POT-derived data revealed the deformation values in the azimuth and LOS directions for the ascending tracks to be −13–25 and −10–39 m, respectively. By correlating the deformation distribution with the regional topography, the azimuth deformation data identified 139 coseismic geohazards. In contrast, the LOS deformation data pinpointed 110. When 93 duplicate identifications were excluded, the total comprised 156 geohazards, categorized as 5 glacier landslides, 27 high mountain rupture geohazards, and 124 coastal landslides. For the descending tracks, the deformation values were noted as −10–15 m in the azimuth direction and −12–20 m in the LOS direction. The azimuth deformation data for this track discerned 81 coseismic geohazards, whereas the LOS data revealed 63. After excluding 55 duplicates, the final count stood at 89 geohazards, which encompassed 3 glacier landslides, 22 high mountain rupture geohazards, and 64 coastal landslides. Cumulatively, based on the InSAR data from both tracks, 245 geohazards were determined (Table 4).

### 5.3. Coseismic Geohazard Verification

To ensure the reliability of the aforementioned geohazard identification, a quantitative assessment was executed on the coseismic geohazard identification derived from the ascending- and descending-track POT results (Table 4). Subsequent to this evaluation, high-resolution optical remote sensing images from the post-earthquake epicenter area were gathered to corroborate the authenticity of the geohazard identifications.

**Figure 9:**
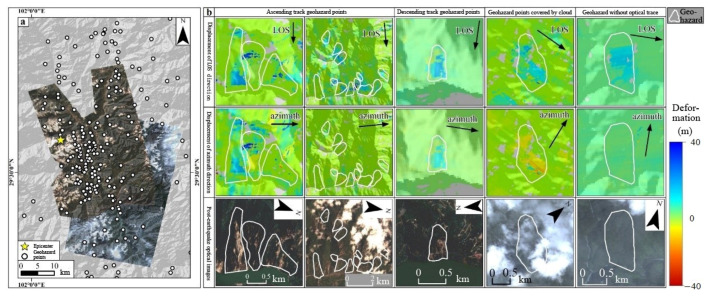
Verification of the detected coseismic geohazard based on optical remote sensing images. (**a**) Coverage of the optical remote sensing images; (**b**) verification of typical geohazards. The yellow star and black circles filled with white in (**a**) show the epicenter and geohazard points, respectively. The white closure lines in (**b**) denote the geohazard areas and the color of these areas denote the deformation magnitude.

The acquired high-resolution optical images from the post-earthquake epicenter are presented in Figure 9a. Significant landform alterations, as depicted in the post-earthquake optical images, served as the benchmark (Figure 9b) for evaluating the efficacy of SAR-POT for geohazard identification purposes (Table 5 and Table 6). The verification rate for the optical images was computed as the ratio of the number of verified geohazard risks to the total number of risks within the optical image’s domain. The analyses indicate verification rates of 71.2% and 64.7% for ascending- and descending-track POT values, respectively. The pinpointed geohazards aligned commendably with the extant optical images (Figure 9b). Notably, unverified points were predominantly localized in the cloud-covered sections of the optical images (Figure 9b). Additionally, as the post-earthquake SAR images were procured subsequent to the optical images, certain geohazard locales could have originated from aftershock events following the capture of optical images (Figure 9b). Nevertheless, it warrants mentioning that discrepancies in some damage points unverified by optical images could have resulted from mismatches during the SAR-POT data processing stage.

### 5.4. Analysis of Detected Coseismic Hazards

To elucidate the types and distribution of the geohazards, quantitative analyses were conducted for the identified coseismic geohazards (Table 7). This analysis was augmented by an exhaustive assessment of the geohazard distribution, factoring in the regional slope aspect, distribution density, fault slip magnitude, and other pertinent variables.

**Figure 10:**
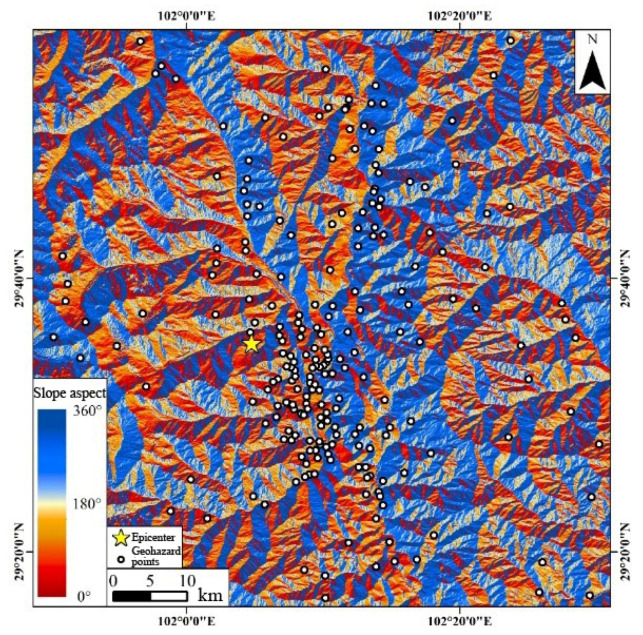
Relationship between slope aspect and geohazard distribution in the study region. The yellow star denotes the epicenter, the black circles filled with white indicate the geohazard points, and the colors in the figure show the slope aspect.

Based on the data presented in Table 7, it is evident that the majority of geohazards precipitated by the coseismic event are coastal landslides, comprising over 71%. This predominance is intrinsically linked to the earthquake’s epicenter proximity to the bank of the Dadu River in Moxi Town. The analysis indicated that the geohazard identification repetition rates for both the ascending and descending tracks in azimuth and LOS directions were around 60%, demonstrating a notable consistency. Additionally, given the seismogenic fault’s alignment was approximately north–south, the concealed coseismic geohazards resultant from the fault predominantly exhibited an east–west orientation (Figure 10). Consequently, when contrasted with the azimuth deformation data, which predominantly captured north–south movements, the LOS POT results—more attuned to detecting east–west and vertical deformations—proved to be more efficacious for identifying the geohazards instigated by the earthquake, boasting a recognition rate exceeding 89%.

**Figure 11:**
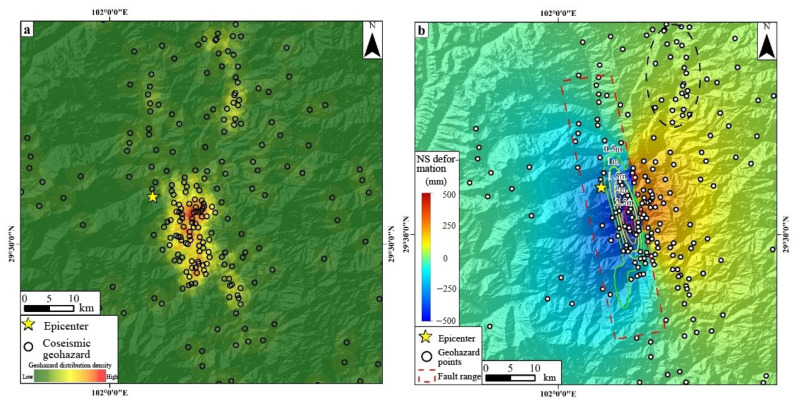
Relationship between geohazard density and fault activity. (**a**) Density of coseismic geohazard distribution. (**b**) Relationship between geohazard distribution and fault activity. The yellow star denotes the epicenter. The black hollow dots in (**a**) show the coseismic geohazard and the colors indicate its distribution density. The black circles filled with white in (**b**) denote the geohazard points, the red dashed rectangle indicates the fault range, the color solid closure lines represent the contour lines, the color in the figure represents the deformation magnitude, and the black dashed ellipse represents the concentrated geohazard distribution in the northeastern footwalls of the fault along the Dadu River.

To reveal the relationship between the coseismic geohazards and seismogenic fault of the Luding earthquake, an examination of the hidden point density of the identified coseismic geohazards was performed. This involved an integrated assessment of the interrelation between the geohazard distribution, coseismic deformation field, and fault data. Figure 11 illustrates that the coseismic geohazards are predominantly distributed southeast of the epicenter (Figure 11a), an area also marked by significant earthquake damage. Further evaluations indicated that the distribution of the coseismic geohazards aligned with the magnitude of the surface deformation. The causative Xianshuihe fault is characterized as a left-lateral strike-slip fault, with its principal movement directed north–south. The north–south-oriented deformation depicted in Figure 11b highlights the density of the coseismic geohazards in regions experiencing pronounced surface deformation magnitudes. Moreover, Figure 11b’s depiction of coseismic fault sliding isolines reveals that geohazards are situated flanking the salient fault rupture sections, with geohazards being more prevalent in the hanging wall zone than in the footwall vicinity proximate to the fault. However, it is imperative to highlight the concentrated geohazard distribution on the northeastern footwalls of the fault along the Dadu River (black dashed ellipse in Figure 11b), potentially resulting from the reservoir bank slope instability induced by the Luding earthquake [42,43].

## 6. Conclusions

In the present study, the coseismic deformation and fault motion parameters of the 2022 Luding Mw 6.8 earthquake were determined using Sentinel-1 satellite SAR images. Concurrently, the POT technique was employed for the identification of coseismic geohazards caused by the earthquake. It was found that the seismogenic fault of the Luding Mw 6.8 earthquake was proximate to the Moxi fault within the Xianshuihe fault zone. The maximum surface deformation in the LOS direction for both the satellite’s ascending and descending tracks measured ~190 mm and ~140 mm, respectively. This deformation distribution suggested that the earthquake was controlled by a predominant left-lateral strike-slip rupture. Additionally, the estimated optimal strike and dip angles for the Luding earthquake fault were 169.3° and 70°, respectively. The coseismic slip was predominantly located at a depth of 3–12 km, with a peak slip of 2.67 m at a depth of 7 km. Notably, the identified coseismic geohazards predominantly spanned both flanks of the significant slip segment on the seismogenic fault, directly correlating with the magnitude of the surface deformation. In conclusion, the notable instability of the bank slopes at the fault’s footwall can be attributed to the seismic activities of the Luding earthquake. 

## Figures and Tables

**Table 1:** Parameters of Sentinel-1A SAR data.

Flight Direction	Tracks	Main Image Data (Day-Month-Year)	Secondary Image Data (Day-Month-Year)	Time Baseline (d)	Vertical Baseline (m)
Ascending	26	26-08-2022	07-09-2022	12	−202.423
Descending	135	02-09-2022	14-09-2022	12	−50.421

**Table 2:** Comparison of seismic fault inversion parameters.

Parameter Source	Strike Angle (°)	Dip Angle (°)	Average Rake Angle (°)	Mw
USGS	345	88	17	6.64
GCMT	163	80	8	6.70
This paper	169.3	70	−8.5	6.72

**Table 3:** Comparison of coseismic slip results.

Comparison Objects	Character	Mw	Fault Rupture Range	Strike Angle	Dip Angle	Seismogenic Fault Length	Maximum Slip Magnitude/Depth
This paper	Left-lateral strike	6.72	3–12 km	169.3°	70°	~21 km	2.67 m/7 km
Li Yanchuan et al. (2022) [33]	Left-lateral strike	6.74	<10 km	162°	79°	~30 km	1.8 m/7 km
Han Bingquan et al. (2023) [34]	Left-lateral strike	6.59	0–10 km	167°	72°	~30 km	2.23 m/5.8 km

**Table 4:** Statistical table of geohazard identifications using the pixel offset tracking technique.

POT Result Type	POT of Ascending Counts of Geohazards	POT of Ascending Counts of Geohazards
Azimuth	139	81
LOS	110	63
Repetition	93	55
Single track	156	89
Both tracks	245	

**Table 5:** Statistical table of geohazard identifications in the remote sensing images.

POT Result Type	POT of Ascending Counts of Geohazards	POT of Ascending Counts of Geohazards
Out of range	52	38
Within range	104	51
Verified	74	33
Covered by cloud	17	12
No obvious trace	13	6

**Table 6:** Geohazard identification verification rates in remote sensing images.

Verification Type	Ascending POT (%)	Descending POT (%)
Verified	71.2	64.7
Covered by cloud	16.3	23.5
No obvious trace	12.5	11.8

**Table 7:** Proportion of different types of geohazards.

Geohazard Identification Type	Ascending POT (%)	Descending POT (%)
Glacier slides	3.2	3.4
High mountain rupture	17.3	24.7
Coastal landslides	79.5	71.9
LOS direction	89.1	91.0
Azimuth direction	70.5	70.7
Repetitions in LOS and azimuth directions	59.6	61.7

## Data Availability

Sentinel-1A satellite SAR images can be downloaded from the European Space Agency (ESA, https://scihub.copernicus.eu/dhus/, accessed on 20 September 2022). The optical images were obtained by the Sichuan Earthquake Administration using an unmanned aerial vehicle (UAV). The extracted InSAR deformation, the estimated faulting model, and the identified landslide dataset was applied by the first and corresponding authors.
